# Characterization of the Temperature-Sensitive Mutations *un-7* and *png-1* in *Neurospora crassa*

**DOI:** 10.1371/journal.pone.0010703

**Published:** 2010-05-18

**Authors:** Michael G. Dieterle, Aric E. Wiest, Mike Plamann, Kevin McCluskey

**Affiliations:** 1 Fungal Genetics Stock Center, School of Biological Sciences, University of Missouri- Kansas City, Kansas City, Missouri, United States of America; 2 Pembroke Hill School, Kansas City, Missouri, United States of America; University of Sevilla, Spain

## Abstract

The model filamentous fungus *Neurospora crassa* has been studied for over fifty years and many temperature-sensitive mutants have been generated. While most of these have been mapped genetically, many remain anonymous. The mutation in the *N. crassa* temperature-sensitive lethal mutant *un-7* was identified by a complementation based approach as being in the open reading frame designated NCU00651 on linkage group I. Other mutations in this gene have been identified that lead to a temperature-sensitive morphological phenotype called *png-1*. The mutations underlying *un-7* result in a serine to phenylalanine change at position 273 and an isoleucine to valine change at position 390, while the mutation in *png-1* was found to result in a serine to leucine change at position 279 although there were other conservative changes in this allele. The overall morphology of the strain carrying the *un-7* mutation is compared to strains carrying the *png-1* mutation and these mutations are evaluated in the context of other temperature-sensitive mutants in Neurospora.

## Introduction

While the genetic map of the filamentous fungus *N. crassa* contains over 1,200 markers [1], the genome sequence predicts closer to 10,000 genes for this organism [2]. Most Neurospora gene deletion mutants show no visible phenotype further emphasizing that classical mutational analysis alone does not allow one to identify every gene in a filamentous fungus [3]. One category of mutant that was available for classical genetic analysis was temperature-sensitive (TS) lethal mutants. For *Neurospora crassa*, numerous TS mutants were generated in the late 1960's and early 1970's [4]. These mutants were called “unknown” to emphasize the fact that the reason for their irremediable temperature-sensitive lethal phenotype was not known. In Neurospora over 50 such mutants were generated and made available to the community via the Fungal Genetics Stock Center [5]. Because a strain carrying an anonymous TS mutation has relatively little value in an era when genome sequencing has largely replaced genetic mapping, we have endeavored to identify mutations underlying several anonymous TS lethal mutations in Neurospora. Schmidhauser *et al* [6] identified overlapping cosmids that complemented one such mutant, *un-7* but did not identify either the open reading frame (ORF) containing the *un-7* mutation or the actual change in the DNA sequence. In this report, we have demonstrated that the ORF mutated in *un-7* is related to a yeast gene whose product is implicated in the targeting of misfolded proteins for degradation by the proteosome, although recent studies have challenged this role in Neurospora [7], [8]. If UN-7 is involved in protein quality control it adds to the observation that many TS lethal mutants in Neurospora are involved in protein biology. For example, two TS lethal mutants are known to affect protein synthesis by impairing ribosome function [9], [10]. Another TS lethal mutant, *un-10*, has a defect in the Neurospora ortholog of the eukaryotic translation initiation factor 3, subunit B [11]. Finally, the TS lethal mutant *un-4* has been shown to carry a mutation in a gene from the mitochondrial protein import pathway [12]. These mutants were selected for characterization based on their genetic location and not because of any similarity of phenotype. Thus a relatively random selection of TS lethal mutants has led to a group of mutants that impact various steps of the protein synthesis, trafficking, and quality control suggesting that this might be a common characteristic of the TS mutants in Neurospora generated by Inoue and colleagues [4]. This is in stark contrast to the distribution of other TS mutations in Neurospora which affect a variety of biological functions, including TS auxotrophs and TS morphological mutants. The characterization of both TS lethal and TS morphological mutations in the same gene in Neurospora emphasizes the value of traditional mutant hunts.

## Results

**Figure 1:**
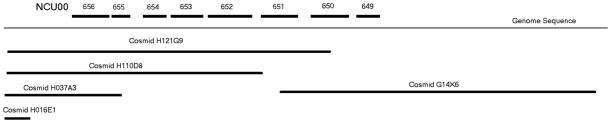
Selected cosmids spanning the genomic region including NCU00651. Open reading frames are indicated above the line representing the genome sequence while the cosmids are indicated below the line.

**Figure 2:**
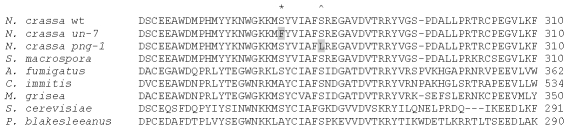
Alignment of the putative amino acid sequence from the mutated region of the NCU00651 protein from wild type and two mutants as well as select fungi. The altered amino acid residues in the UN-7 and PNG-1 proteins are indicated by grey shading and amino acid residue 273 is indicated with an “*” above the sequence. Amino acid residue 279 is indicated with a “∧” above the sequence. The locus designations for other species are as follows: *Sordaria macrospora* CBI51252; *Aspergillus fumigatus* EDP54057; *Coccidioides immitis* CIMG_08062; *Magnaporthe grisea* MGG_03598; *Saccharomyces cerevisiae* EE09111; *Phycomyces blakesleeanus* 178891.

Schmidhauser *et al* [6] previously identified cosmids that complemented *un-7*, but did not identify the gene defined by the *un-7* mutation. The Neurospora genome sequencing program sequenced the ends of clones from the same cosmid library that Schmidhauser and colleagues used allowing us to directly evaluate these results in the context of the genome sequence [2]. As the overlap of these cosmids did not allow us to identify the mutated ORF directly, we used multiple approaches to identify the ORF that is mutated in the *un-7* strain including complementation with additional overlapping cosmids and complementation by PCR products corresponding to genes contained in the genomic region spanned by the cosmids that successfully complemented the mutation in strains carrying *un-7* (Figure 1). When cells carrying the ts-lethal mutant allele *un-7* were transformed with cosmid H121G9 from the pLORIST6xh library (Table 1), they regained the ability to grow at 37°C (Table 2). Cosmid H121G9 includes eleven open reading frames. Cosmids overlapping H121G9, (Figure 1 and Table 2) were evaluated for their ability to restore wild-type growth to cells carring the TS-lethal allele *un-7* at 37°C. None of these other cosmids complemented the *un-7* mutation efficiently suggesting that one of the ORFs unique to H121G9 was *un-7^+^*. PCR products from ORFs in the region of minimum overlap (NCU00651- NCU00655) were tested for their ability to complement the *un-7* mutation and only NCU00651 was successful (Table 2). Therefore we conclude that the ORF which is mutated in strains carrying *un-7* is NCU00651. Since this ORF was identified in 2003 as being mutated in a TS morphological mutant called *png-1* [7], we obtained DNA sequence for both *un-7* and for *png-1* allele 22-9. These two mutants appear to be different alleles of the same gene. When compared to the wild-type sequence there were several changes in each DNA sequence and these corresponded to both conservative and non-conservative changes in the amino acid sequences. In *un-7* there was a serine to phenylalanine change at position 273(T to C at base 817) and an isoleucine to valine change at position 390 in the amino acid sequence (A to G at base 1168). The sequence of *png-1* allele 22-9 included a serine to leucine change at position 279 in the amino acid sequence (C to T at base 836). The changes at positions 273 and 279 are in positions that are highly conserved among other organisms (Figure 2).

**Table 1:** Strains and plasmids used in this work.

Strain or plasmid	Characteristics	Reference
FGSC 2489	Wild type	2
FGSC 2175	*un-7 mat-A*	4
FGSC 2176	*un-7 mat-a*	4
FGSC 9860	*png-1 mat-a*	7
pMDFGSC651w	NCU00651 PCR product amplified from FGSC 987 genomic DNA cloned into pSC-A-amp/kan (TCAAACAGGCGACACAGG to TGCATGCCTCCTTTCCTT)	This study
pMDFGSC651c	NCU00651 PCR product amplified from cosmid pLORIST6xh H121G9 cloned into pSC-A-amp/kan	This study
pLORIST6xh H121G9	Supercontig 1: 7650088-7695847*, ORFs NCU00651 - NCU00662	16
pLORIST6xh H110D8	Supercontig 1: 7648553-7689801, ORFs NCU00652 - NCU00663	16
pLORIST6xh H037A3	Supercontig 1: 7640433-7677263, ORFs NCU00655 - NCU00665	16
pLORIST6xh H016E1	Supercontig 1: 7629706-7669107, ORFs NCU00658 - NCU00667	16
pLORIST6xh H011C11	Supercontig 1: 7619903-7660589, ORFs NCU00659 - NCU00670	16
pMOcosX G14 C6	Supercontig 1: 7691511-7724894, ORFs NCU00635 - NCU00650	16

*Refers to Assembly 10.

**Table 2:** Complementation of the mutation *un-7*.

Transforming DNA^a^	Strain # (number of replicates)	Colonies at 37°C	HygR colonies at room temperature b
**Protoplasts**			
pLORIST6xh H121G9	2176 (1)	28	31
pLORIST6xh H121G9	2175 (3)	287	ND
pMOcosX G14C6	2175 (3)	38	20
pMDFGSC651w	2176 (2)	45	ND
pMDFGSC651c	2176 (1)	26	ND
NCU00651 WT PCR	2176 (1)	20	ND
NCU00651 9882 PCR	2176 (4)	41	ND
No-DNA control c	2175 (1)	4	0
No-DNA control	2176 (2)	12	0
**Electroporation**			
pLORIST6xh H121G9	2175 (2)	54	ND
pLORIST6xh H016E1	2175 (2)	3	>50
pLORIST6xh H037A3	2175 (2)	1	ND
pLORIST6xh H011C11	2175 (2)	1	>100
pLORIST6xh H110D8	2175	1	ND
PCR NCU00651	2175	25	ND
PCR NCU00652	2175	1	ND
PCR NCU00653	2175	0	ND
PCR NCU00654	2175	3	ND
PCR NCU00655	2175	1	ND

Multiple transformations were carried out with each cosmid and the average result is presented for each strain tested.

a In transformation with cosmid DNA, 3 ug of transforming DNA was used for each replicate. For transformation with PCR products, 0.8 to 1 ug of DNA was used for each experiment. DNA concentration was determined using a NanoDrop1000 UV/VIS Spectrophotometer.

b Hygromycin phosphotransferase is carried on the pLORIST6xh and pMOcosX cosmids. This test was included as a control but was not possible with PCR products or genes cloned into pSC-A-amp/kan.

c Colonies on no DNA control plates are the total number per 10^7^–10^8^ protoplasts.

**Figure 3:**
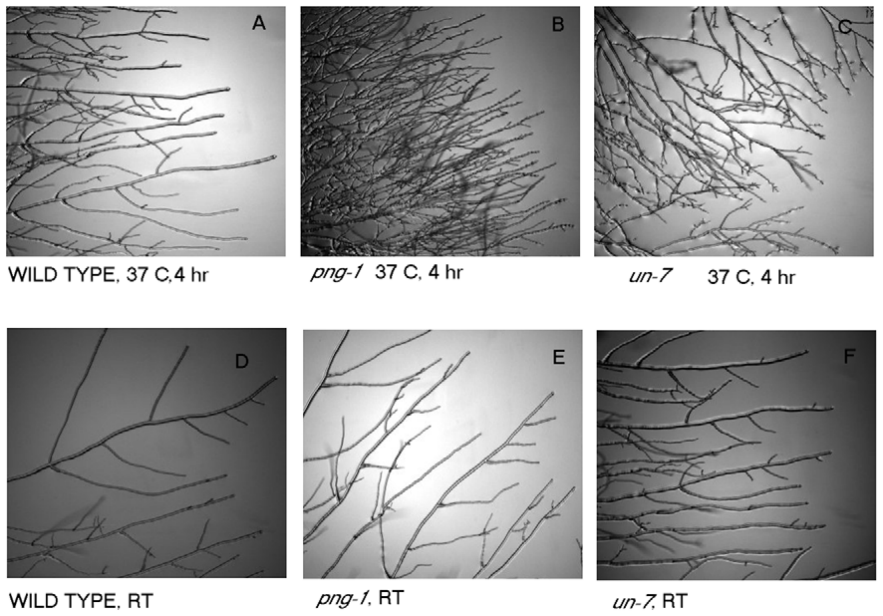
Temperature shift of growing tips of wild-type, *un-7* and *png-1* strains to 37°C. Panels A–C were taken after shifting overnight slide cultures incubated at room temperature to 37°C for 4 hours and panels D–F show the cultures grown on slides at room temperature. (A,D) Wild type strain 2489. (B, E) *png-1* strain 9860. (C, F) *un-7* strain 2176.

To evaluate the sequence differences in a biological context, we tested the ability of strains carrying different alleles of NCU00651 to grow at the restrictive temperature. While the wild-type strain FGSC 2489 grew normally at 37°C, the growth of a strain carrying *png-1* was approximately half that of wild type after both 1.5 hours and 4 hours (Table 3). Growth of a strain carrying the *un-7* mutation dropped to just above one third the wild type rate after 1.5 hours at 37°C and no additional growth occurred after 1.5 hours (Figure 3).

**Table 3:** Cumulative growth of wild type and mutant strains at 37°.

Time (hours)	WT	*un-7*	*png-1*
1.5	4.5+/−0.5 mm	1.7+/−0.3 mm	2.8+/−0.3 mm
4.0	10.7+/−0.3 mm	1.8+/−0.3 mm	5+/−0.3 mm

Values are the average of three measurements plus or minus the standard deviation.

## Discussion

Temperature-sensitive mutations in *Neurospora crassa* occur in a variety of pathways. Of over 100 TS mutations represented by Neurospora strains at the Fungal Genetics Stock Center, 56 are non-remedial TS lethal and 13 are TS morphological mutations. The remaining 36 mutations produce auxotrophies that are capable of being overcome by specific nutrient supplementation [1]. The observation that numerous TS lethal, and not other TS mutants, are involved in protein synthesis, transport, or, as suggested by the current study, protein quality control (Table 4), implies that additional TS lethal mutants generated by Inoue and colleagues in Neurospora [4] may impact protein synthesis pathways.

**Table 4:** Neurospora TS lethal genes involved in protein production, transport, or quality control.

TS Lethal gene	function	Citation
*rip-1*	Ribosomal protein	10
*un-16*	Ribosomal protein S9	9
*un-10*	EIF3b	11
*un-4*	Tim16	12
*un-25*	Ribosomal protein L13	6
*un-7*	Unknown/*png-1*	This study

Complementation of anonymous TS lethal mutations with their wild-type sequence allows one to use selection at 37°C to identify the ORF carrying the mutation. This approach has led to the identification of NCU00651 as the gene historically known as *un-7* [4]. Complementation with cosmid DNA was more efficient than with the amplified NCU00651 ORF, possibly because the PCR fragment contained only 53 bases 5′ of the start site. The original PCR product was designed to include 140 upstream bases and 104 downstream bases, but changes in annotation of the 5′ end of this ORF occurred after these experiments were complete. Because the Neurospora functional genomics program [3] makes significant data available online it is possible to examine the expression of this gene in a number of different contexts. Transcription analyses comparing strains of different mating types, in synthetic heterokaryons, or in response to H_2_O_2_ treatment show no significant difference in gene expression for the wild type allele of NCU00651 [13]. Additional analyses showed that transcription of NCU00651 was strongly induced by phytosphingosine, an inducer of programmed cell death [13], [14]. Together these results show that NCU00651 is transcribed during normal growth, and that transcriptional regulation of this gene occurs in response to external stimuli. Other alleles of NCU00651 have been identified as giving rise to a temperature-sensitive morphological phenotype [7]. Although the screen used by Seiler and Plamann identified morphological mutations in NCU00651 with high frequency, their screen was not designed to find TS lethal mutations. Morover, the Neurospora functional genomics program produced a deletion of NCU00651, but was unable to purify it as a homokaryon, suggesting that the gene is essential [3].

Thus, different alleles of the same ORF have resulted in different phenotypes and this is clearly seen in the difference in linear growth when strains carrying different alleles of NCU00651 are exposed to the restrictive temperature. The changes in morphology seen when shifting strains carrying the *un-7* allele and the *png-1* allele to 37°C are similar for the first hour after which strains carrying the *un-7* allele stop growing completely. The difference in the deficit in linear growth between *un-7* and *png-1* (Table 3) suggests that the *un-7* allele is more deleterious than the *png-1* allele. Both carry mutations that encode a nonconservative changes and both changes are in the region of the protein that is proposed to interact with *rad-23* (NCU07542), a protein suggested to be involved in nucleotide excision repair [15].

Both *un-7* and *png-1* have additional conservative changes in their amino acid sequence. The changes in the PNG-1 putative protein include an aspartic acid to asparagine at position 60 and a glutamic acid to lysine change at position 313. The only additional change in the UN-7 putative protein is an isoleucine to valine at position 390. In the *N. tetrasperma* and *N. discreta* genes, all of these positions are identical with the wild-type *N. crassa* sequence. Interestingly, both *N. tetrasperma* and *N. discreta* have other conservative changes in their NCU00651 orthologs (not shown). Comparison of the mutated region of the UN-7 and PNG-1 protein sequence with the same region from other filamentous fungi shows the strong conservation of the mutated residues in other organisms. Only Coccidioides and Phycomyces do not share the conserved serine at the position corresponding to position 273 in the Neurospora sequence. Similarly only Saccharomyces does not have a serine at the position corresponding to 279 in the Neurospora sequence (Figure 2).

PNG1 proteins in other systems are implicated in shunting mis-folded or aberrant proteins to the proteosome for degradation [15]. Maerz *et al* [8] showed that the Neurospora PNG-1 protein does not have N-glycanase activity and compared the growth of either *png-1* or *rad-23* mutants with the growth of a strain carrying both mutations. The double mutant had signficantly reduced linear growth compared to either mutation alone reiterating that there is some combined function [8]. This study also investigated the affect of various inhibitory drugs on strains carrying *png-1* and concluded that the role of the UN-7/PNG-1 protein was neither in wall synthesis nor in protein turnover. Thus, the role of the UN-7/PNG-1 protein in protein turnover in Neurospora, and by analogy in other filamentous fungi is still undefined. The demonstration that the UN-7/PNG-1 protein does not have N-glycanase activity challenges the association between this protein and protein quality control and raises the possibility that the lethality of the *un-7* mutation is related to the binding of its protein product to RAD23 and their subsequent involvement in DNA repair.

Characterization of additional TS-lethal mutations in Neurospora will help to clarify the relationship between protein synthesis, transport and quality control and natural or induced mutants in these processes.

## Materials and Methods

Standard molecular genetic techniques were used for the preparation and analysis of nucleic acids. Cosmids from the pLORIST6xh or pMOcosX libraries [16] were propagated in *E. coli* DH5 alpha. PCR products were cloned into the vector pSC-A-amp/kan (Stratagene, La Jolla CA). Transformation of *N. crassa* was carried out using polyethylene glycol fusion of protoplasts [17] or electroporation of intact conidia [18]. For the protoplast based transformations, the top agar was prepared using low gelling temperature agarose instead of Bacto Agar allowing plating to be carried out a lower temperature. Selection for cosmids or PCR products that complement the *un-7* mutation was carried out by placing the transformation plates at 37°C. Colonies were apparent after two to four days. Selection for hygromycin resistance was carried out at room temperature [19]. DNA sequencing was performed by the University of Missouri- Kansas City, School of Biological Sciences Genomics facility using subcloned fragments amplified from genomic DNA and deposited in Genbank (*un-7* Genbank accession #1321877, *png-1* allele 22-9 Genbank accession #1321867). Photo microscopy was carried out on an Olympus BX50 microscope with a Diagnostic Instruments RT SE 12.1 monochrome digital imaging system. Specimens for microscopy were prepared by culturing on a glass microscope slide coated with Vogels minimal medium [20]. Conidia were spotted on one end of the microscope slide and allowed to grow overnight. The slides were held in petri dishes at room temperature and were kept in a closed plastic box in the presence of several wet paper towels to maintain high humidity. Temperature stress was induced by moving the box to a 37°C incubator for the time indicated.
